# Enhancing Performance of Continuous-Variable Quantum Key Distribution (CV-QKD) and Gaussian Modulation of Coherent States (GMCS) in Free-Space Channels under Individual Attacks with Phase-Sensitive Amplifier (PSA) and Homodyne Detection (HD)

**DOI:** 10.3390/s24165201

**Published:** 2024-08-11

**Authors:** Nancy Alshaer, Tawfik Ismail, Haitham Mahmoud

**Affiliations:** 1 Department of EEC, Faculty of Engineering, Tanta University, Tanta 31527, Egypt; 2 National Institute of Laser Enhanced Sciences, Cairo University, Giza 12613, Egypt; tismail@nu.edu.eg; 3 Department of Telecommunication Engineering, Taibah University, Medina P.O. Box 344, Saudi Arabia; 4 Faculty of Computing, Engineering and Build Environment, Birmingham City University, Birmingham B4 7XG, UK

**Keywords:** continuous-variable quantum key distribution (CV-QKD), Gaussian modulation of coherent states (GMCS), free space quantum channels, atmospheric turbulence, secret key rate (SKR), phase-sensitive amplifier (PSA), homodyne detection, individual attack

## Abstract

In recent research, there has been a significant focus on establishing robust quantum cryptography using the continuous-variable quantum key distribution (CV-QKD) protocol based on Gaussian modulation of coherent states (GMCS). Unlike more stable fiber channels, one challenge faced in free-space quantum channels is the complex transmittance characterized by varying atmospheric turbulence. This complexity poses difficulties in achieving high transmission rates and long-distance communication. In this article, we thoroughly evaluate the performance of the CV-QKD/GMCS system under the effect of individual attacks, considering homodyne detection with both direct and reverse reconciliation techniques. To address the issue of limited detector efficiency, we incorporate the phase-sensitive amplifier (PSA) as a compensating measure. The results show that the CV-QKD/GMCS system with PSA achieves a longer secure distance and a higher key rate compared to the system without PSA, considering both direct and reverse reconciliation algorithms. With an amplifier gain of 10, the reverse reconciliation algorithm achieves a secure distance of 5 km with a secret key rate of $10^{-1}$ bits/pulse. On the other hand, direct reconciliation reaches a secure distance of 2.82 km.

## 1. Introduction

Quantum key distribution (QKD) is a popular technique that gives two parties (Alice and Bob) the ability to construct a shared cryptographic secret key [1,2,3]. This technique relies on the principles of quantum mechanics and the Heisenberg uncertainty principle to ensure the security of the key exchange, making it resistant to eavesdropping attacks [4]. QKD has been widely studied and implemented in various communication systems, including fiber-optic networks and satellite-based communication systems. The QKD protocols are classified into two main categories: discrete variable (DV-QKD) and continuous variable (CV-QKD) [5]. DV-QKD protocols use single photons to transmit information, while CV-QKD protocols use coherent states of light. The well-known standard DV-QKD protocols are BB84 [6], B92 [7], and SARG04 [8]. On the other hand, the popular standard protocols for CV-QKD are presented by Ralph [9], Hillery [10], and Grosshans and Grangier [11]. Recent studies show the continuous development of DV-QKD and CV-QKD [12,13,14,15,16,17,18,19,20,21].

This article focuses on utilizing free space as the transmission medium, taking into account the challenges posed by atmospheric attenuation and turbulence. Both of these factors can negatively impact the performance and reliability of QKD systems operating in free-space communication [22,23]. Furthermore, using single-photon DV-QKD has solidly proven the effectiveness of quantum protocols previously tested only in limited terrestrial experiments. It has been shown that it is highly robust against channel noise compared to CV-QKD, making it particularly suitable for low-loss (low-noise) channels [24]. On the other hand, the CV-QKD regime is perhaps most closely related to classical wireless communications and holds the potential to enhance communication performance, especially in the highly lossy channels addressed in this study [25]. Therefore, implementing DV-QKD in a free-space communication scenario becomes significantly more complex and costly compared to CV-QKD due to the unique challenges posed in such an environment. Furthermore, the CV-QKD systems can convey more information per signal than DV-QKD. However, increasing the transmission distance in the CV-QKD system is one of the most important aspects of carrying out a security-proof assessment. This is because the performance of CV-QKD systems is more sensitive to losses and noise, which can significantly affect the quality of the quantum signals. Therefore, optimizing a CV-QKD system for a given transmission distance is crucial for achieving secure and efficient communication [26]. One approach to optimize a CV-QKD system for a given transmission distance is to incorporate amplifiers, which can amplify the quantum signals [27]. Another approach is to improve the quality of the quantum signals by using advanced modulation [28] and reconciliation techniques [29].

CV-QKD over a lossy channel with transmittance and excess noise is a challenging problem due to the impact of noise and losses on the quality of the transmitted quantum signal [30,31]. In addition, the presence of excess noise in the channel can introduce additional errors and limit the achievable key rate. This can make it difficult to establish a secure key between the two parties, especially over long distances or in harsh environments. In order to overcome the challenges of the secret key rate (SKR) in QKD systems, several techniques have been developed, including optical preamplifiers [32,33], error correction [34], privacy amplification [35], advanced modulation [36,37], and encoding schemes [38,39]. These techniques aim to enhance the overall performance and reliability of the transmission process. These techniques can help to improve the robustness and reliability of CV-QKD over lossy channels with transmittance and excess noise, making it possible to establish secure communication between the two parties.

In CV-QKD, it is important to analyze its security against possible attacks [40,41]. One of the most significant sources of noise and disturbance in CV-QKD is the loss of quantum states during transmission through the communication channel. This loss limits Eve’s ability to perform a collective attack, as the signal-to-noise ratio decreases with the distance of the channel [42]. Furthermore, in a realistic collective attack, Eve requires considerable time and/or coherent operations to decode the stored ensemble and approach the Holevo information collectively. Therefore, the feasibility and success of a collective attack depend on the available resources and capabilities of Eve [43]. In this context, an individual attack becomes the optimal eavesdropping attack for the no-switching/homodyne detection protocol (the attacker cannot switch or swap quantum states during the transmission process) [44]. This attack occurs when Eve prepares her probe states individually, couples them to the signal states, and then immediately measures them individually [45]. It allows Eve to steal some information about the signal states without disturbing them, which limits the ultimate security of quantum communication. The individual attack can be prevented using the switching protocol, where Alice and Bob randomly switch between different measurement bases to prevent Eve from gaining complete information about the signal states. Significant research has focused on applying individual attacks to QKD systems, resulting in satisfactory security performance [43,46,47,48,49]. In order to considerably improve the system performance, including the secret key rate (SKR) and propagation range, it is necessary to ensure the protocol security merely against individual attacks. Furthermore, to improve performance and security drawbacks, post-amplifier and reconciliation techniques, as well as modulation and detection mechanisms, could be integrated within CV-QKD systems [50,51,52]. This integration can lead to the development of more reliable and secure systems. Therefore, further research is required to optimize these components and achieve the practical implementation of CV-QKD for secure communication over long distances.

The main contribution of this study is to propose a system model for a CV-QKD system which employs the Gaussian modulation coherent states (GMCS) protocol. This system model is specifically designed to address the challenges associated with free-space quantum channels, particularly in the presence of individual attacks using a phase-sensitive amplifier (PSA) and homodyne detection (HD). By developing this system model, the study aims to improve the security and efficiency of quantum communication over long distances. This contribution is essential for enhancing the reliability and robustness of CV-QKD systems under individual attacks, paving the way for the practical implementation and optimization of quantum cryptography for secure long-distance communications.

The remainder of this work is divided into the following sections. The atmospheric quantum channel model is investigated over HD in Section 2. A general individual attack scenario then assesses the CV-QKD secret key rate. The system security is analyzed in Section 3, and the numerical evaluation of the system performance is given in Section 4. Finally, the conclusions are presented in Section 5.

## 2. Channel and System Models

This section presents the model of the atmospheric quantum channel considering weak turbulence. The block diagram of a prepare and measure-based GMCS-CVQKD protocol with HD will be discussed, assuming a general individual attack scenario.

### 2.1. Space Quantum Channel Model

Channel transmittance is a key measure of the quality of a communication channel. It refers to the fraction of the transmitted signal received by the receiver after it has traveled through the channel. It can impact the ability of the receiver to detect and interpret the transmitted signal accurately. The quality of the channel’s transmittance can be affected by various factors, such as attenuation, noise, and distortion. The probability distribution of the transmission coefficient (PDTC) for the atmospheric channel in the free-space quantum communication is used efficiently to describe the optical beam propagation through the turbulent atmosphere. The PDTC model differs depending on the turbulence severity, distinguished through Rytov variance values [31]. The general model of the PDTC in [31] assumes that a Gaussian elliptical beam propagates across an air channel characterized by isotropic turbulence, where it experiences wandering, broadening, and shape deformation into an elliptical. In a weak turbulence regime where beam-wandering losses are dominant, the distribution of the transmission coefficient simplifies to the log-negative generalized Rice distribution. If the beam fluctuates around the aperture center, this distribution reduces to the log-negative Weibull distribution [30]. On the other hand, the beam broadens and deforms for the weak-to-moderate transition and strong turbulence, producing a smooth PDTC with a more problematic evaluation compared to the weak turbulence case [31,53].

In this work, it is assumed that (1) beam deflection is due to the imprecise adjustment of the radiation source; (2) the beam incidence is approximately normal to the aperture plane; and (3) a Gaussian beam is traveling through free space with a spot radius of *W* at the receiver end.

**Figure 1:**
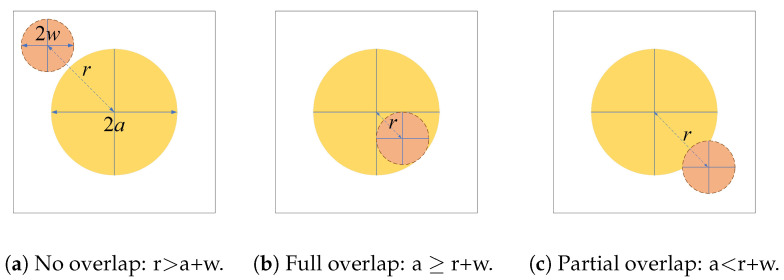
Overlap scenarios between the beam spot (dashed circle) and the photodetector plan (solid circle).

Given that the beam center undergoes a deflection of distance (*r*) from the aperture center, three scenarios can be identified regarding the projections of the optical beam spot with radius (*W*) on the photodetector plan with radius (*a*) as illustrated in Figure 1. The magnitude of the intersecting area between the beam spot and the photodetector plane will directly impact the amount of optical power that the photodetector can capture.

The incomplete Weber integral is employed to calculate the transmission efficiency $T^{2}$ of a Gaussian beam propagating through the atmospheric channel as [30]:

(1) $$T^{2}=\frac{2}{\pi W^{2}}e^{-2\frac{r^{2}}{W^{2}}}\int_{0}^{a}\;d\varrho \varrho \;e^{-2\frac{r^{2}}{W^{2}}}I_{0}\left(\frac{4}{W^{2}}r\varrho \right)\;,$$

where $I_{n}\left[.\right]$ denotes the modified Bessel function of the first kind of the *n*-th order. In channels characterized by fluctuating loss, the transmission coefficient *T* is a real and positive random variable. Furthermore, it is implied that T∈[0,1] to preserve the commutation relations. An approximate analytical expression of (1) is proposed in [30] and given by:

(2) $$T^{2}=T_{0}^{2}\exp\left[{\left(\frac{-r}{R}\right)}^{\Gamma }\right],$$

where $T_{0}$ is the maximum value of *T*, *R* is the scale parameter, and Γ is the shape parameter. These three parameters are described as follows [30]:

(3) $$T_{0}^{2}=1-\exp\left[-2{\left(\frac{a}{w}\right)}^{2}\right]\;,$$

(4) $$R=a{\left[\ln\left(\frac{2T_{0}^{2}}{1-\exp\left[-{\left(\frac{2a}{w}\right)}^{2}\right]I_{0}\left[{\left(\frac{2a}{w}\right)}^{2}\right]}\right)\right]}^{-\frac{1}{\Gamma }}\;,\;$$

(5) $$\Gamma =8{\left(\frac{a}{w}\right)}^{2}\frac{\exp\left[-{\left(\frac{2a}{w}\right)}^{2}\right]I_{1}\left[{\left(\frac{2a}{w}\right)}^{2}\right]}{1-\exp\left[-{\left(\frac{2a}{w}\right)}^{2}\right]I_{0}\left[{\left(\frac{2a}{w}\right)}^{2}\right]}\times {\left[\ln\left(\frac{2T_{0}^{2}}{1-\exp\left[-{\left(\frac{2a}{w}\right)}^{2}\right]I_{0}\left[{\left(\frac{2a}{w}\right)}^{2}\right]}\right)\right]}^{-1}.$$

The spot radius *W* of the received beam, with optical wavelength λ, is calculated as [54]:

(6) $$W=\sqrt{W_{o}^{2}+\xi {\left(\frac{\lambda d}{\pi W_{o}}\right)}^{2}}\;,$$

where $\xi =1+(2W_{o}^{2}/\rho_{o}^{2})$, $\rho_{o}={\left(0.55\;C_{n}^{2}\;k^{2}\;d\right)}^{-\frac{3}{5}}$ is the coherence length, k=2π/λ is the optical wave number, the beam spot radius at Alice is $W_{o}$, and the air link distance from Alice to Bob is *d*. For the weak to strong turbulence conditions, the value of the refractive index structure parameter $C_{n}^{2}$ varies from $10^{-17}$ $m^{-2/3}$ to $10^{-13}$ $m^{-2/3}$ [54].

**Figure 2:**
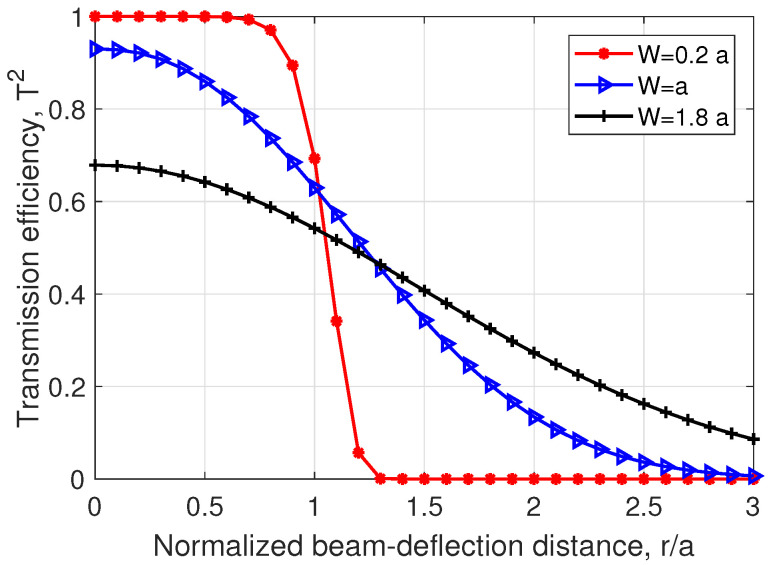
Transmission efficiency versus normalized beam-deflection distance for different beam spot radii.

Figure 2 illustrates the relationship between the transmission efficiency $T^{2}$ and the normalized beam-deflection distance r/a, considering various ratios of the received beam spot radius *W* relative to the detector radius *a*. By analyzing the graph, we can understand how the efficiency of transmission is influenced by the distance between the transmitter and receiver. This information is valuable in analyzing and optimizing CV-QKD systems in applications where these parameters play a significant role.

### 2.2. Mathematical Foundations of the Main Processes

The mathematical formulations provide a detailed foundation for understanding the processes involved in the GMCS protocol, homodyne detection, reconciliation algorithms, and the integration of PSA with homodyne detection.

(1) **GMCS Protocol:** The GMCS protocol is a fundamental method in CV-QKD systems, using coherent states of light modulated with Gaussian distributions to encode information in the quadratures of the light. In GMCS, the amplitude and phase of coherent light pulses are modulated with Gaussian-distributed random variables, defining the quantum states. This allows for efficient and high-dimensional encoding of quantum information in a continuous variable space [55,56]:
  (7) $$x∼N\left(0,\;V_{m}\right),p∼N\left(0,\;V_{m}\right)$$
  where *x* and *p* are the quadrature of the coherent states |α〉 with modulation variance $V_{m}$ and mean zero.
(2) **Homodyne Detection:** Once the coherent states are modulated, they are transmitted through the quantum channel to the receiver. At the receiver’s end, techniques such as homodyne detection are employed to measure the quadratures of the received coherent states. Homodyne detection measures the quadrature of the optical field as [57]
  (8) $${\hat{x}}_{\theta }=\hat{x}\cos\theta +\hat{p}\sin\theta$$
  where θ is the phase difference between the signal and the local oscillator. The output quadrature ${\hat{x}}_{out}$ measured by the detector is related to the input quadrature ${\hat{x}}_{in}$ and the vacuum noise quadrature ${\hat{x}}_{vac}$ as follows:
  (9) $${\hat{x}}_{out}=\sqrt{\eta }{\hat{x}}_{in}+\sqrt{1-\eta }{\hat{x}}_{vac}$$
  where η represents the detection efficiency. The total noise in the homodyne detection process combines both the detection inefficiency and the electronic noise $\nu_{el}$. It is given by:
  (10) $$\nu_{det}=\frac{1}{\eta }+\nu_{el}$$
(3) **Direct Reconciliation (DR) and Reverse Reconciliation (RR):** DR and RR are two essential techniques used for error correction and privacy amplification. These techniques are important for ensuring that the quantum key generated between the sender (Alice) and the receiver (Bob) is identical, secure, and free from potential eavesdropping. In DD, the process starts with Alice sending her encoded quantum states to Bob through the quantum channel. Bob then measures the received states. In RR, the roles of Alice and Bob in the reconciliation process are reversed. The secure key length is given by [58]
  (11) $$\begin{array}{c} K_{DR}=I\left(A;B\right)-I\left(A;E\right)\;\;\;\;\mathrm{Direct}\;\mathrm{Reconciliation}\;\;\\ K_{RR}=I\left(B;A\right)-I\left(B;E\right)\;\;\;\;\mathrm{Reverse}\;\mathrm{Reconciliation} \end{array}$$
  where I(A;B), I(A;E), I(B;A), and I(B;E) are the mutual information between Alice and Bob, Alice and Eve, Bob and Alice, and Bob and Eve, respectively.
(4) **Integration of PSA and HD:** The integration of PSA with HD is used to enhance the performance of CV-QKD systems. PSA can amplify quantum signals while preserving their noise properties, making them particularly useful for compensating for detector imperfections and enhancing the overall detection process. The output quadratures after the amplification are given by [59]
  (12) $${\hat{x}}_{out}=\sqrt{g}{\hat{x}}_{in}\;\;\;;\;\;\;{\hat{p}}_{out}=\frac{{\hat{p}}_{in}}{\sqrt{g}}$$
  where *g* is the amplification gain. The total detection noise after PSA compensation can be expressed as [60]:
  (13) $$\nu_{out}=\frac{\nu_{det}\left(1-\eta \right)}{g\eta }$$

### 2.3. System Model

**Figure 3:**
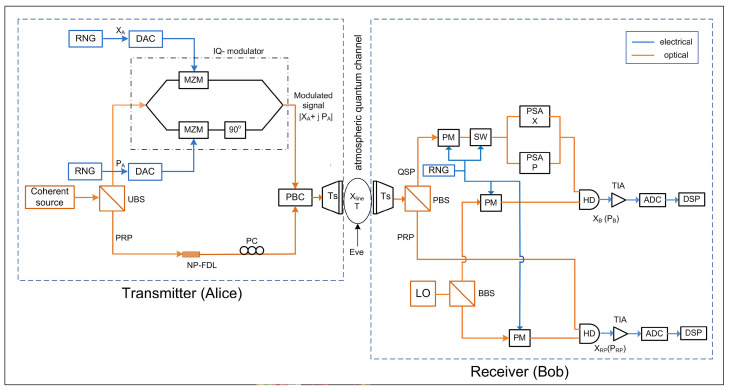
Schematic of a prepare and measure GMCS-CVQKD protocol, under individual attack, over atmospheric fading channel with HD and PSA. Eve intercepts the channel transmittance and excesses the noise, but she cannot access Bob’s detection equipment. RNG: random number generator; DAC: digital-to-analog converter; UBS: unbalanced beam splitter; MZM: Mach–Zehnder modulator; PBC: polarized beam combiner; NP-FDL: nonpolarized-fiber delay line; PC: polarization controller; PRP: phase reference pulse; PSA: phase-sensitive amplifier; PBS: polarized beam splitter; BBS: balanced beam splitter; PM: phase modulator; SW: switch; HD: homodyne detector; TIA: transimpedance amplifier; ADC: analog-to-digital converter; DSP: digital signal processor.

Figure 3 shows the schematic of a prepare and measure GMCS-CVQKD protocol over a free-space fading channel. A coherent optical source is used at the transmitter (Alice) to produce a train of coherent state pulses that are separated using an unbalanced beam splitter (UBS). Alice randomly chooses two values for the orthogonal quadrature $P_{A}$ and the in-phase quadrature $X_{A}$ for every signal pulse. This is achieved using two independent random number generators (RNGs) that produce zero-mean Gaussian distributed random variables (RVs) *N*, i.e., $N∼N(0,\;V_{A})$ where $V_{A}$ is Alice’s modulation variance. An in-phase-quadrature (IQ) modulator modulates the weak optical pulses, using the Gaussian RVs to introduce quantum signal pulses (QSPs). Now, Alice has finished preparing the QSPs of GMCS $|X_{A}+iP_{A}|$. On the other hand, the relatively stronger pulses are delayed to produce the phase reference pulses (PRPs). Then, she combines the QSPs and the PRPs into orthogonal polarization modes employing a polarization beam combiner (PBC) and transmits them to Bob using the transmitter telescope over the atmospheric quantum channel that has a transmittance *T* and noise $X_{\mathrm{line}}$.

At the receiver, a polarization beam splitter (PBS) separates the time-polarization multiplexing pulses: the QSPs and the PRPs. Bob utilizes homodyne detection (HD) for these two pulses. The required local oscillator (LO) is locally generated and is split using a balanced beam splitter (BBS) to accomplish coherent detection. The HD depends on measuring one of the two quadratures, either *X* or *P*, at random, i.e., not simultaneously. For this purpose, an RNG controls the phase modulators (PMs). A phase-sensitive amplifier (PSA) is useful for HD since it ideally enables noiseless amplification of the selected quadrature [60]. The transimpedance amplifier (TIA) at the receiver front end amplifies the current, detected quadrature, at the HD output to an acceptable voltage level to drive the analog-to-digital converter (ADC). Alice and Bob have now completed the quantum states’ transmission via a feasibly insecure atmospheric quantum channel. Finally, Alice’s modulated data and Bob’s measurement results are utilized to establish a trusted secure key during the classical post-processing procedure over an authenticated public channel. This procedure includes two main steps, a reconciliation process to retrieve an identical sequence of bits from the correlated data and a typical privacy amplification approach to distilling a finalized secret key from this sequence.

## 3. Security Analysis

This section explains and analyzes the usage of the PSA to improve the performance of the homodyne detector model and hence boost the system SKR. Section 3.1 discusses mathematically how the noise performance of the homodyne detector can be modified using the PSA. In Section 3.2, we provide a detailed analysis of the proposed system security under an Eve individual attack in an atmospheric channel for both direct and reverse reconciliation algorithms considering the properties of the free-space channel.

### 3.1. Compensation for Homodyne Detector Noise

In the proposed system, Bob is assumed to always amplify the quadrature that he has selected at random to measure. When the PSA is applied to compensate for the detector imperfections, the quadratures are asymmetrically affected by the amplification process, with the in-phase one amplified and the orthogonal one squeezed. If *x* is the target quadrature to be amplified, the amplification process can be represented as $x\to \sqrt{g}x$, $p\to 1/\sqrt{g}p$, where *g* is the PSA gain, and for any value of *g* greater than one, the target quadrature is amplified. Accordingly, the detection-added noise for the homodyne detector is given as [60]:

(14) $$X_{\mathrm{hom}}^{\mathrm{PSA}}=\frac{\left(1-\eta \right)+v_{el}}{g\eta }$$

where the efficiency of a practical detector is η and the electronic noise variance is $v_{el}$ in shot noise units (SNUs). From (14), it is clear that the amplifier gain will compensate for the finite detector efficiency, hence reducing the detection noise and improving the secret key rate as will be numerically discussed in Section 4.

### 3.2. Secret Key Rate under Individual Attack

In an individual attack, Eve executes an independent and identically distributed (i.i.d.) attack on all pulses. This means that she creates separable ancilla states, each interacting individually with one coherent-state pulse sent by Alice in the quantum channel. She uses a quantum memory to store her states. After the sifting procedure, in HD, after Bob has exhibited the quadrature he selects to measure, she performs her measurements before the reconciliation process. Information reconciliation, defined as correcting errors while minimizing the information revealed to Eve, can be direct or reversed. In DR, Bob corrects his bits according to Alice’s data, while in RR, Alice corrects her bits according to Bob’s data [57]. This subsection deduces the proposed system security for both DR and RR.

#### 3.2.1. Direct Reconciliation

In the direct reconciliation (DR) scenario, Alice’s sequence is used as the target to correct Bob’s sequence. The expression for the DR secret key rate in [58] is modified to incorporate the free-space channel study and reconciliation efficiency. It is deduced as follows:

(15) $$\begin{array}{cc} \Delta I_{DR} & =\left(1-P\right)\;\left[\beta I_{AB}-I_{AE}\right]\\ \; & =\frac{\left(1-P\right)}{2}\left[\beta \log_{2}\left(\frac{V_{A}}{V_{A|B}}\right)-\log_{2}\left(\frac{V_{A}}{V_{A|E}}\right)\;\right], \end{array}$$

where *P* is the link interruption probability caused by the angle-of-arrival fluctuations, for the case of weak turbulence considered in this article (P≈0) [53]. β is the reconciliation efficiency, $I_{AB}$ is the mutual information between Alice and Bob, and the mutual information between Alice and Eve is $I_{AE}$. $V_{A}$ is Alice’s modulation variance. The conditional variance between Alice and Bob is $V_{A|B}$; it denotes the variance in Alice’s quadrature, either $X_{A}$ or $P_{A}$, when measured by Bob. $V_{A|E}$ is the conditional variance between Alice and Eve, and it represents the variances in Eve’s estimates of Bob’s field quadratures. Both variances should be minimized by Bob and Eve, respectively. These variances are expressed as [58]:

(16) $$V_{A|B}=\frac{\left(VX+1)(V+X\right)}{2(V+X)}.$$

(17) $$V_{A|E}=\frac{\left(VX+1)(V+X\right)}{2(VX+1)},$$

$X=X_{\mathrm{line}}+X_{\mathrm{hom}}^{\mathrm{PSA}}/T$ is the total noise referred to the channel input and $V=V_{A}+1$. Regarding the channel input, the total channel-added noise is defined in SNU as $X_{\mathrm{line}}=1/T-1+\epsilon$ [23], where ϵ is the free-space channel excess noise measured at the channel input.

Substituting (16) and (17) into (15), the DR secret key rate is formulated as follows:

(18) $$\Delta I_{DR}=\frac{1}{2}\log_{2}\left[{\left(\frac{2(V-1)}{\left(VX+1)+(V+X\right)}\right)}^{\beta -1}\times \left(\frac{{\left(V+X\right)}^{\beta }}{\left(VX+1\right)}\right)\right]\;,$$

In perfect reconciliation, β = 1, the secret key rate reaches its maximum value as:

(19) $$\Delta I_{DR,\max}=\frac{1}{2}\log_{2}\left(\frac{V+X}{VX+1}\right)\;,$$

#### 3.2.2. Reverse Reconciliation

In the reverse reconciliation (RR) scenario, to correct Alice’s sequence, Bob’s sequence is used as the target. The expression for the RR secret key rate in [58] is similarly modified to incorporate the study of the free-space channel and reconciliation efficiency. It is deduced as follows:

(20) $$\begin{array}{cc} \Delta I_{RR} & =\left(1-P\right)\;\left[\beta I_{BA}-I_{BE}\right]\\ \; & =\frac{\left(1-P\right)}{2}\left[\beta \log_{2}\left(\frac{V_{B}}{V_{B|A}}\right)-\log_{2}\left(\frac{V_{B}}{V_{B|E}}\right)\right]\;, \end{array}$$

where $I_{BA}=I_{AB}$ is the mutual information between Bob and Alice, $I_{BE}$ is the mutual information between Bob and Eve, $V_{B|A}$ is the conditional variance between Bob and Alice, $V_{B|E}$ is the conditional variance between Bob and Eve, and $V_{B}$ is Bob’s modulation variance. They are accordingly expressed as:

(21) $$V_{B|A}=T\left(1+X\right)\;,$$

(22) $$V_{B|E}=\frac{1}{T(V^{-1}+X)}\;,$$

(23) $$V_{B}=T\left(V+X\right)\;,$$

by substituting (21), (22), and (23) into (20), the expression of the RR secret key rate is obtained as:

(24) $$\Delta I_{RR}=\frac{1}{2}\log_{2}\left[\frac{1}{{\left(V+X\right)}^{1-\beta }{\left(1+X\right)}^{\beta }T^{2}\left(V^{-1}+X\right)}\right]\;,$$

In perfect reconciliation, β = 1, the secret key rate reaches its maximum value as:

(25) $$\Delta I_{RR,\max}=\frac{1}{2}\log_{2}\left(\frac{1}{\left(1+X\right)T^{2}\left(V^{-1}+X\right)}\right)\;.$$

#### 3.2.3. Signal-to-Noise Ratio (SNR)

In CV-QKD, the signal-to-noise ratio (SNR) is important because it affects the key rate that can be achieved over the channel. A higher SNR generally leads to a higher key rate, but this comes at the cost of increased power consumption and more complex modulation and detection techniques. As presented in (26), the choice of the modulation variance is important in order to determine the amount of noise that the signal can tolerate before it becomes too degraded to extract the secret key [61]. A higher modulation variance can increase the SNR but also make the signal more vulnerable to noise and losses in the channel:

(26) $$\mathrm{SNR}=\frac{V_{A}}{\left(1+X\right)}$$

## 4. Numerical Results and Discussion

This section presents the numerical results that evaluate the impact of using the PSA in conjunction with the realistic homodyne detector of the system that was proposed in Section 2. These results are based on the analysis and derivations that were discussed in Section 4. For the representation of the GMCS/CVQKD protocol under individual attack in free space, the SKR is calculated in terms of the propagation range considering the channel transmittance and excess noise for DR and RR algorithms. All simulation parameters are summarized in Table 1. MathWorks-MATLAB-2021A is utilized for the simulation.

**Figure 4:**
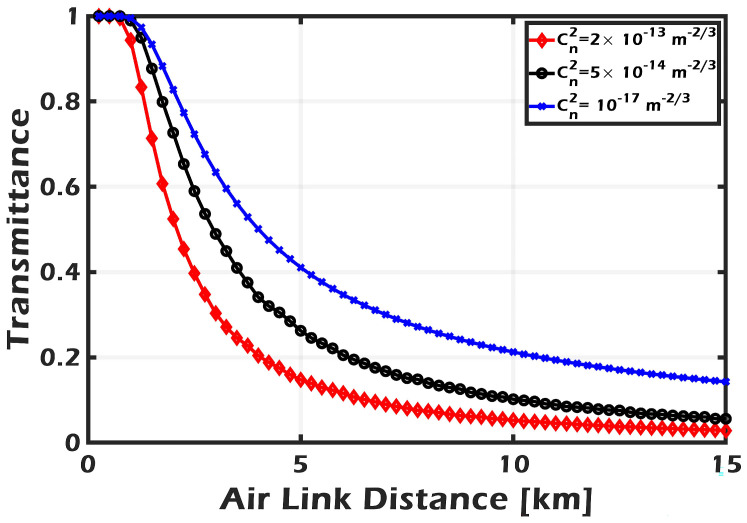
Transmittance of the free-space channel versus air link distance for different turbulence regimes.

**Figure 5:**
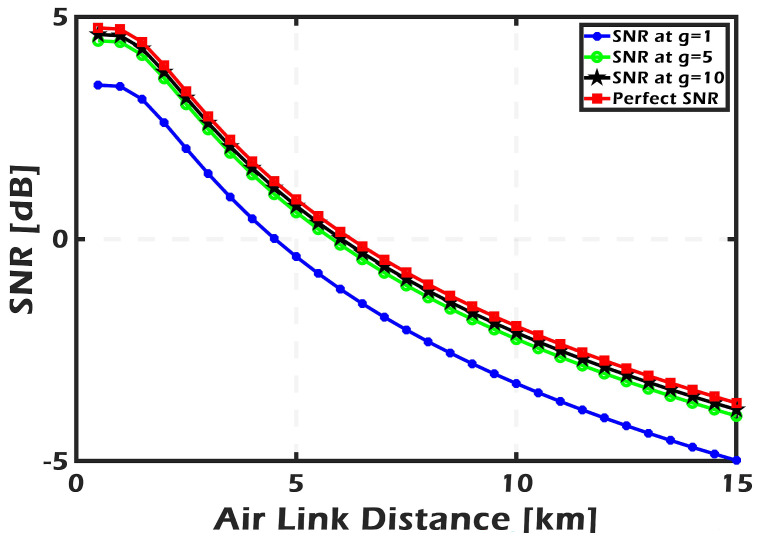
The behavior of SNR with different transmission distances as a function of the PSA gain.

The transmittance of the space quantum channel is introduced in Figure 4 as a decreasing of the beam propagation distance for different atmospheric turbulence cases, including weak ($10^{-17}$), moderate ($5\times 10^{-14}$), and strong ($2\times 10^{-13}$). For a distance smaller than 1 km, the transmittance is almost unaffected by turbulence variations and is greater than 0.9. The fluctuation in turbulence noticeably affects the transmittance over greater distances. For example, as the distance increased from 5 km to 10 km, the transmittance decreased by half as the turbulence changed from weak to strong. According to the beam size and aperture radius that were used in the simulation, the transmittance reached its half maximum at distances of 4 km, 3.5 km, and 2.6 km for weak, moderate, and strong turbulence, respectively. Those variations in the transmittance will be reflected in the SKR. In the simulation, the amplifier gain is set to the following values: 1 (representing the performance without an amplifier), 5, and 10. The perfect case is called perfect detection with zero noise, i.e., 100% detector efficiency, zero electronic noise, and no amplification. Figure 5 shows the improvement in the received SNR due to applying the PSA amplifier. As it can be seen, the SNR approach is very close to its perfect value when the amplifier gain g=5 or 10.

**Figure 6:**
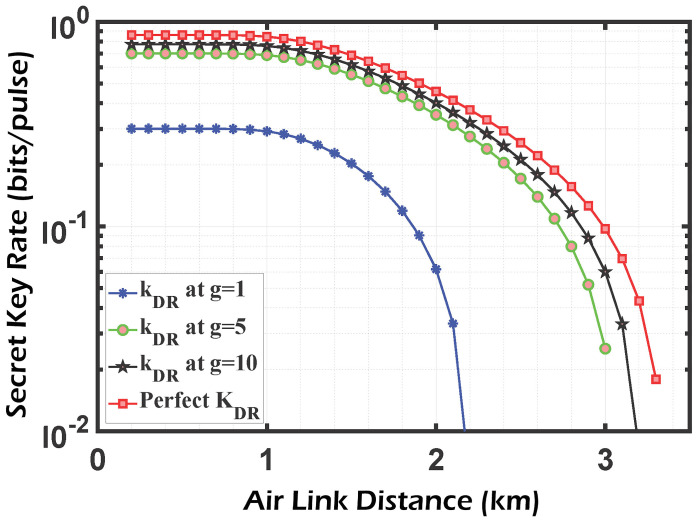
SKR vs. channel distance for a protocol using HD and PSA with DR, the ’perfect secret key’ corresponds to a perfect homodyne detector ($\eta =1,v_{el}=0,\;g=1$).

**Figure 7:**
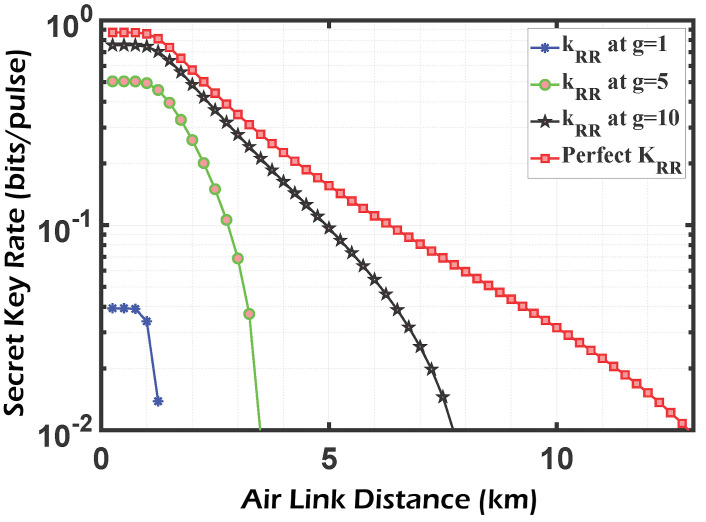
SKR vs. channel distance for a protocol using HD and PSA with RR, the ’perfect secret key’ corresponds to a perfect homodyne detector ($\eta =1,v_{el}=0,\;g=1$).

In (14), the homodyne detector adds noise after connecting the PSA, which tends to zero when the amplification gain is really high. As a result, an ideal PSA compensates for all imperfections in a practical homodyne detector, making their combination equivalent to a flawless detector from a system point of view. This approach is illustrated numerically in Figure 6 and Figure 7, where the SKR line at g=10 approximates the ideal detector line. In addition, the effects of the free-space channel transmittance, as well as excess noise, are taken into consideration.

**Figure 8:**
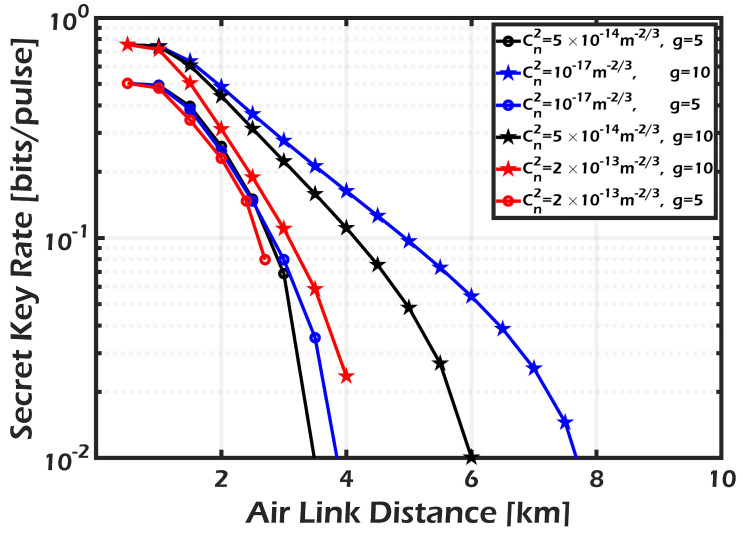
SKR vs. channel distance in RR for different turbulence conditions using PSA with g=5 and g=10.

Figure 6 and Figure 7 show the secret key rate of the CV-QKD/GMCS system for the direct reconciliation and reverse reconciliation methods, respectively. The results demonstrate that the DR technique outperforms the RR approach in terms of both the gained SKR and the achievable transmission distance. These observations are made before accounting for the impact of the phase-sensitive amplifier (PSA) with a gain value of g=1 as indicated by the blue curve. As discussed in [11], DR is preferred over RR when dealing with noisy channels. This preference aligns with our system’s case, which does not incorporate PSA. The state is inverted when the PSA is coupled with the realistic homodyne detector, and the performance of the RR technique exceeds that of the DR, especially considering the propagation distance. When the amplifier gain g=10, an SKR of $10^{-2}$ bits/pulse can be sent over a distance of 7.8 km in the RR technique compared to only 3.2 km in the DR technique. Those figures also show that for RR, without the PSA, the maximum distance is limited to 1 km, which satisfies an SKR of $3.5\times 10^{-2}$ bits/pulse. After coupling the PSA with gain equals 10, this distance is increased 5 times (5 km), corresponding to an increase of approximately 3 times ($10^{-1}$) in the achievable SKR. On the other hand, the limited distance of 1 km (corresponding to an SKR of $3\times 10^{-1}$) for the DR algorithm is only doubled (2 km) after applying the PSA of gain 10, and its corresponding SKR ($4\times 10^{-1}$) is multiplied by a factor of 1.3 compared to the case without an amplifier. It can be concluded that RR introduces better performance in terms of the allowable transmission distance than DR when applying the PSA. The results shown in the previous figures are calculated under a weak turbulence regime ($C_{n}^{2}=10^{-17}$ $m^{-2/3}$). Next, we will evaluate the effect of turbulence variation on the system security using RR with the following values for the refractive index structure parameter $C_{n}^{2}$: $10^{-17}$ $m^{-2/3}$, $5\times 10^{-14}$ $m^{-2/3}$, and $2\times 10^{-13}$ $m^{-2/3}$ corresponding to weak, moderate, and strong turbulence respectively. It is worth noting that the PSA of gain =10, as opposed to that of gain =5, is very effective when considering the turbulence effect, where longer transmission distances are attained as the severity of the turbulence decreases as shown in Figure 8.

## 5. Conclusions

In this article, the performance of a GMCS/CV-QKD system is evaluated over a free-space optical channel under a general individual attack. This article assumes homodyne detection followed by either direct or reverse reconciliation mechanisms. The transmittance of the atmospheric quantum channel is considered, as well as its variation with the propagation distance. This article aimed to investigate the impact of atmospheric turbulence on the performance of quantum key distribution systems. The results show that the transmittance of the channel decreased with distance, leading to a reduction in the secure key rate. Employing a PSA to compensate for the finite efficiency of the homodyne detectors improves the achievable SKR and the available secure propagation range. The results reveal that comparing the system performance before and after using the PSA, in the case of the RR technique, the improvement in the system performance after coupling a PSA (with a gain of 10) in terms of the SKR and transmission distance is approximately 2.5 times that for the case of the DR. Therefore, it can be concluded that the use of PSA can significantly enhance the performance of homodyne-based QKD systems and that the RR technique is more sensitive to the use of PSA than the DR technique. However, further studies are needed to investigate the impact of amplifier compensation under other attacks, such as collective and coherent attacks.

## Figures and Tables

**Table 1:** Simulation parameters; all variances and noises are in SNUs [23,54,60,62].

Parameter	Description	Value
*a*	Aperture radius	0.15 m
$W_{o}$	beam-spot radius	0.005 m
λ	Laser wavelength	1550 nm
$V_{A}$	Alice’s modulation variance	4
ϵ	Excess noise	0.005
$v_{el}$	Electronic noise	0.01
η	Detector efficiency	0.75
*g*	Amplifier gain	1, 5, and 10
*d*	Propagation distance	0–15 km
$C_{n}^{2}$	Refractive index structure parameter	$10^{-17}$, $5\times 10^{-14}$, and $2\times 10^{-13}$$m^{-2/3}$

## Data Availability

There is no data associated with this publication.

## References

[1] Xu F., Ma X., Zhang Q., Lo H.K., Pan J.W. (2020). Secure quantum key distribution with realistic devices. Rev. Mod. Phys..

[2] Alshaer N., Ismail T. (2024). AI-Driven Quantum Technology for Enhanced 6G networks: Opportunities, Challenges, and Future Directions. J. Laser Sci. Appl..

[3] Renner R., Wolf R. (2023). Quantum advantage in cryptography. AIAA J..

[4] Ali M.Z., Abohmra A., Usman M., Zahid A., Heidari H., Imran M.A., Abbasi Q.H. (2023). Quantum for 6G communication: A perspective. IET Quantum Commun..

[5] Morris J.D., Grimaila M.R., Hodson D.D., Jacques D., Baumgartner G. (2014). A survey of quantum key distribution (QKD) technologies. Emerging Trends in ICT Security.

[6] Bennett C.H., Brassard G. An update on quantum cryptography. Proceedings of the Workshop on the Theory and Application of Cryptographic Techniques.

[7] Bennett C.H. (1992). Quantum cryptography using any two nonorthogonal states. Phys. Rev. Lett..

[8] Scarani V., Acin A., Ribordy G., Gisin N. (2004). Quantum cryptography protocols robust against photon number splitting attacks for weak laser pulse implementations. Phys. Rev. Lett..

[9] Ralph T.C. (1999). Continuous variable quantum cryptography. Phys. Rev. A.

[10] Hillery M. (2000). Quantum cryptography with squeezed states. Phys. Rev. A.

[11] Grosshans F., Van Assche G., Wenger J., Brouri R., Cerf N.J., Grangier P. (2003). Quantum key distribution using gaussian-modulated coherent states. Nature.

[12] Alshaer N., Nasr M.E., Ismail T. (2021). Hybrid MPPM-BB84 quantum key distribution over FSO channel considering atmospheric turbulence and pointing errors. IEEE Photonics J..

[13] Ramos M.F., Pinto A.N., Silva N.A. (2022). Polarization based discrete variables quantum key distribution via conjugated homodyne detection. Sci. Rep..

[14] Primaatmaja I.W., Liang C.C., Zhang G., Haw J.Y., Wang C., Lim C.C.W. (2022). Discrete-variable quantum key distribution with homodyne detection. Quantum.

[15] Alshaer N., Moawad A., Ismail T. (2021). Reliability and security analysis of an entanglement-based QKD protocol in a dynamic ground-to-UAV FSO communications system. IEEE Access.

[16] Fan-Yuan G.J., Lu F.Y., Wang S., Yin Z.Q., He D.Y., Chen W., Zhou Z., Wang Z.H., Teng J., Guo G.C. (2022). Robust and adaptable quantum key distribution network without trusted nodes. Optica.

[17] Wang H., Li Y., Pi Y., Pan Y., Shao Y., Ma L., Zhang Y., Yang J., Zhang T., Huang W. (2022). Sub-Gbps key rate four-state continuous-variable quantum key distribution within metropolitan area. Commun. Phys..

[18] Alshaer N., Ismail T., Nasr M.E. (2020). Performance evaluation and security analysis of ground-to-satellite FSO system with CV-QKD protocol. IET Commun..

[19] Ruiz-Chamorro A., Garcia-Callejo A., Fernandez V. (2024). Low-complexity continuous-variable quantum key distribution with true local oscillator using pilot-assisted frequency locking. Sci. Rep..

[20] Ding S., Shen G., Tang F., Chan C.C.K. (2024). Noise-aware resource allocation with integrated key generation and consumption for CV-QKD over WDM networks. J. Opt. Commun. Netw..

[21] Alshaer N., Ismail T. (2022). Performance evaluation and security analysis of UAV-based FSO/CV-QKD system employing DP-QPSK/CD. IEEE Photonics J..

[22] Li M., Wang T. (2020). Continuous-variable quantum key distribution over air quantum channel with phase shift. IEEE Access.

[23] Chai G., Cao Z., Liu W., Wang S., Huang P., Zeng G. (2019). Parameter estimation of atmospheric continuous-variable quantum key distribution. Phys. Rev. A.

[24] Lasota M., Filip R., Usenko V.C. (2017). Robustness of quantum key distribution with discrete and continuous variables to channel noise. Phys. Rev. A.

[25] Hosseinidehaj N., Babar Z., Malaney R., Ng S.X., Hanzo L. (2018). Satellite-based continuous-variable quantum communications: State-of-the-art and a predictive outlook. IEEE Commun. Surv. Tutor..

[26] Lopez-Leyva J.A., Talamantes-Alvarez A., Ponce-Camacho M.A., Garcia-Cardenas E., Alvarez-Guzman E. (2018). Free-Space-Optical Quantum Key Distribution Systems: Challenges and Trends. Quantum Cryptography in Advanced Networks.

[27] Li Y., Wang Y., Mao Y., Peng W., Jin D., Guo Y. (2021). Continuous-Variable Quantum Key Distribution Based on Heralded Hybrid Linear Amplifier with a Local Local Oscillator. Entropy.

[28] Pan Y., Wang H., Shao Y., Pi Y., Li Y., Liu B., Huang W., Xu B. (2022). Experimental demonstration of high-rate discrete-modulated continuous-variable quantum key distribution system. Opt. Lett..

[29] Mao H.K., Li Q., Hao P.L., Abd-El-Atty B., Iliyasu A.M. (2022). High performance reconciliation for practical quantum key distribution systems. Opt. Quantum Electron..

[30] Vasylyev D.Y., Semenov A., Vogel W. (2012). Toward global quantum communication: Beam wandering preserves nonclassicality. Phys. Rev. Lett..

[31] Vasylyev D., Semenov A., Vogel W. (2016). Atmospheric quantum channels with weak and strong turbulence. Phys. Rev. Lett..

[32] Qi J., Peng J., Liu W., He C., Zhang M. (2021). Performance improvement of self-referenced continuous-variable quantum key distribution via optical amplifiers. Laser Phys. Lett..

[33] Guo Y., Li R., Liao Q., Zhou J., Huang D. (2018). Performance improvement of eight-state continuous-variable quantum key distribution with an optical amplifier. Phys. Lett. A.

[34] Gümüş K., Eriksson T.A., Takeoka M., Fujiwara M., Sasaki M., Schmalen L., Alvarado A. (2021). A novel error correction protocol for continuous variable quantum key distribution. Sci. Rep..

[35] Tang B.Y., Liu B., Zhai Y.P., Wu C.Q., Yu W.R. (2019). High-speed and large-scale privacy amplification scheme for quantum key distribution. Sci. Rep..

[36] Wang P., Zhang Y., Lu Z., Wang X., Li Y. (2023). Discrete-modulation continuous-variable quantum key distribution with a high key rate. New J. Phys..

[37] Wang H., Pi Y., Huang W., Li Y., Shao Y., Yang J., Liu J., Zhang C., Zhang Y., Xu B. (2020). High-speed Gaussian-modulated continuous-variable quantum key distribution with a local local oscillator based on pilot-tone-assisted phase compensation. Opt. Express.

[38] Vagniluca I., Da Lio B., Rusca D., Cozzolino D., Ding Y., Zbinden H., Zavatta A., Oxenløwe L.K., Bacco D. (2020). Efficient time-bin encoding for practical high-dimensional quantum key distribution. Phys. Rev. Appl..

[39] Pathak N.K., Chaudhary S., Sangeeta, Kanseri B. (2023). Phase encoded quantum key distribution up to 380 km in standard telecom grade fiber enabled by baseline error optimization. Sci. Rep..

[40] Kish S.P., Thapa C., Sayat M., Suzuki H., Pieprzyk J., Camtepe S. (2024). Mitigation of channel tampering attacks in continuous-variable quantum key distribution. Phys. Rev. Res..

[41] Guo Y., Yin P., Huang D. (2023). One-pixel attack for continuous-variable quantum key distribution systems. Photonics.

[42] Wang X., Guo S., Wang P., Liu W., Li Y. (2019). Realistic rate-distance limit of continuous-variable quantum key distribution. Opt. Express.

[43] Hosseinidehaj N., Walk N., Ralph T.C. (2019). Optimal realistic attacks in continuous-variable quantum key distribution. Phys. Rev. A.

[44] Weedbrook C., Lance A.M., Bowen W.P., Symul T., Ralph T.C., Lam P.K. (2004). Quantum cryptography without switching. Phys. Rev. Lett..

[45] Hosseinidehaj N., Walk N., Ralph T.C. (2021). Composable finite-size effects in free-space continuous-variable quantum-key-distribution systems. Phys. Rev. A.

[46] Lütkenhaus N. (2000). Security against individual attacks for realistic quantum key distribution. Phys. Rev. A.

[47] Waks E., Takesue H., Yamamoto Y. (2006). Security of differential-phase-shift quantum key distribution against individual attacks. Phys. Rev. A—Atomic Mol. Opt. Phys..

[48] Rastegin A.E. (2019). Individual attacks with generalized discrimination and inadequacy of some information measures. Quantum Inf. Process..

[49] Ramanathan V., Prabhakar A., Mandayam P. (2023). Security of differential phase shift QKD against explicit individual attacks. arXiv.

[50] Huang P., Huang J., Zhang Z., Zeng G. (2018). Quantum key distribution using basis encoding of Gaussian-modulated coherent states. Phys. Rev. A.

[51] Liang J., Zhou J., Shi J., He G., Guo Y. (2016). Improving Continuous-Variable Quantum Key Distribution Using the Heralded Noiseless Linear Amplifier with Source in the Middle. Int. J. Theor. Phys..

[52] Kundu N.K., McKay M.R., Mallik R.K. (2024). Wireless quantum key distribution at terahertz frequencies: Opportunities and challenges. IET Quantum Commun..

[53] Wang S., Huang P., Wang T., Zeng G. (2018). Atmospheric effects on continuous-variable quantum key distribution. New J. Phys..

[54] Ismail T., Leitgeb E., Ghassemlooy Z., Al-Nahhal M. (2018). Performance improvement of FSO system using multi-pulse pulse position modulation and SIMO under atmospheric turbulence conditions and with pointing errors. IET Netw..

[55] Ahmed S., Alshaer N., Alaghbari K.A., Ismail T. (2022). Security analysis of gaussian and discrete modulations in fso/cv-qkd systems employing llo under phase and amplitude attacks. IEEE Access.

[56] Tang X., Kumar R., Ren S., Wonfor A., Penty R., White I. (2020). Performance of continuous variable quantum key distribution system at different detector bandwidth. Opt. Commun..

[57] Laudenbach F., Pacher C., Fung C.H.F., Poppe A., Peev M., Schrenk B., Hentschel M., Walther P., Hübel H. (2018). Continuous-variable quantum key distribution with Gaussian modulation—The theory of practical implementations. Adv. Quantum Technol..

[58] Niset J. (2008). Quantum Information with Optical Continuous Variables: Nonlocality, Entanglement, and Error Correction. Ph.D. Thesis.

[59] Huang Y., Zhang Y., Xu B., Huang L., Yu S. (2020). A modified practical homodyne detector model for continuous-variable quantum key distribution: Detailed security analysis and improvement by the phase-sensitive amplifier. J. Phys. B At. Mol. Opt. Phys..

[60] Fossier S., Diamanti E., Debuisschert T., Tualle-Brouri R., Grangier P. (2009). Improvement of continuous-variable quantum key distribution systems by using optical preamplifiers. J. Phys. B At. Mol. Opt. Phys..

[61] Li M., Cvijetic M. (2018). Continuous-variable quantum key distribution with self-reference detection and discrete modulation. IEEE J. Quantum Electron..

[62] Farid A.A., Hranilovic S. (2007). Outage capacity optimization for free-space optical links with pointing errors. J. Light. Technol..

